# Immune-Related Gene Variants as Modifiers of Multiple Sclerosis Severity

**DOI:** 10.3390/ijms27125347

**Published:** 2026-06-13

**Authors:** Olga Kulakova, Natalia Baulina, Maxim Kozin, Natalia Matveeva, Alexey Boyko, Olga Favorova, Ivan Kiselev

**Affiliations:** Laboratory of Medical Genomics, Pirogov Russian National Research Medical University, Moscow 117997, Russia; olga.koulakova@gmail.com (O.K.); olga.favorova@gmail.com (O.F.)

**Keywords:** multiple sclerosis, severity, MSSS, genetic polymorphism, immune-related genes, neuroinflammation

## Abstract

Multiple sclerosis (MS) is a heterogeneous autoimmune disorder of the central nervous system of polygenic nature. Uncovering the genetic predictors of MS phenotype can help to explain the nature of the disease’s clinical heterogeneity, and contribute to the development of novel tools for precise disease prognosis. We conducted a retrospective genetic association study of 35 polymorphic variants in immune-related genes with MS severity assessed using the Multiple Sclerosis Severity Score (MSSS) in a sample of 548 Russian relapsing-onset MS patients who have not previously received immunomodulatory therapy. Variants in the *CXCR5*, *EOMES*, *TNFRSF1A*, *IRF8*, *PVT1*, *CCR5*, *HLA-DRB1*, *IL6*, *TCF7*, and *CD40* genes were identified as MSSS-associated in at least two of the three models analyzed (MSSS > 3.5 versus ≤3.5; MSSS > 5.0 versus <2.5; MSSS as a continuous variable). Among them, variants in *CCR5*, *HLA-DRB1* and *IL6* genes were associated with MSSS only in women, while variants in the *TCF7* and *CD40* genes only in men. The variant in *CXCR5* was MSSS-associated both in the total sample and in subgroups of female and male MS patients. Thus, we demonstrate that several GWAS-identified MS risk genes, along with other immunological loci, act as modifiers of the MS phenotype.

## 1. Introduction

Multiple sclerosis (MS) is a common immune-mediated disorder of the central nervous system (CNS) of polygenic nature that leads to neurological disability and is characterized by high clinical heterogeneity [1]. Even within the most common relapsing–remitting course, disease symptoms, relapse severity, duration of remissions, age at onset, and rate of disability progression vary significantly [2]. However, in clinical practice, no widely accepted, validated prognostic model currently exists that can reliably predict clinical outcomes in individuals with MS [3].

The concordance of MS courses in families suggests involvement of genetic variations in modifying the MS phenotype [4,5,6]. The interest in uncovering genetic predictors of MS severity stems from the idea that they can explain the nature of the observed clinical heterogeneity [7] and, at the same time, can contribute to the development of tools for reliable disease prognosis and personalized treatment.

The results of genome-wide association studies (GWAS) obtained in recent years indicated that the genetic architecture underlying MS severity is distinct from that determining disease susceptibility, and pointed to CNS pathways mostly involved in neuronal degeneration and repair [8,9]. However, among CNS pathways both key components of MS pathogenesis, neurodegeneration and neuroinflammation, drive disease progression, contributing to MS severity to varying degrees across different patients [10,11,12].

Given the substantial role of neuroinflammation in MS progression, we conducted in 548 Russian relapsing-onset MS patients a retrospective study of the association between disease severity and polymorphic variants in 35 immune-related genes involved in key stages of innate and adaptive immunity. To estimate MS severity, we used the original global Multiple Sclerosis Severity Score (MSSS) [13], which relates patients’ scores on the Expanded Disability Status Scale (EDSS) to the distribution of disability in patients with comparable disease durations; this version of the MSSS remains the most widely used despite the proposal of several newer versions [14]. An important feature of our study is that it was conducted in treatment-naive patients who had not previously received immunomodulatory drugs, since these agents can substantially alter MSSS by changing the natural course of the disease [15].

Women are affected by MS much more frequently than men, with a female-to-male ratio of 2.3–3.0:1 across different populations. They tend to develop the disease earlier in life but generally respond better to drug therapy. At the same time, men are more likely to experience an aggressive MS clinical course [16]. Taking into account these sex differences in MS prevalence and clinical phenotypes, as well as data on several sex-specific genetic associations with these phenotypes [9,17,18,19], we also performed an association analysis of polymorphic variants in immune-related genes with MSSS separately in women and men.

## 2. Results

Association analysis of genetic variants with MS severity was performed using three different models proposed in [20]: (1) dichotomization of MSSS based on the overall median (median MSSS); (2) extreme phenotype sampling (extreme MSSS); and (3) treating MSSS as a continuous variable (continuous MSSS).

### 2.1. MSSS Distribution in the Studied MS Patients

**Figure 1:**
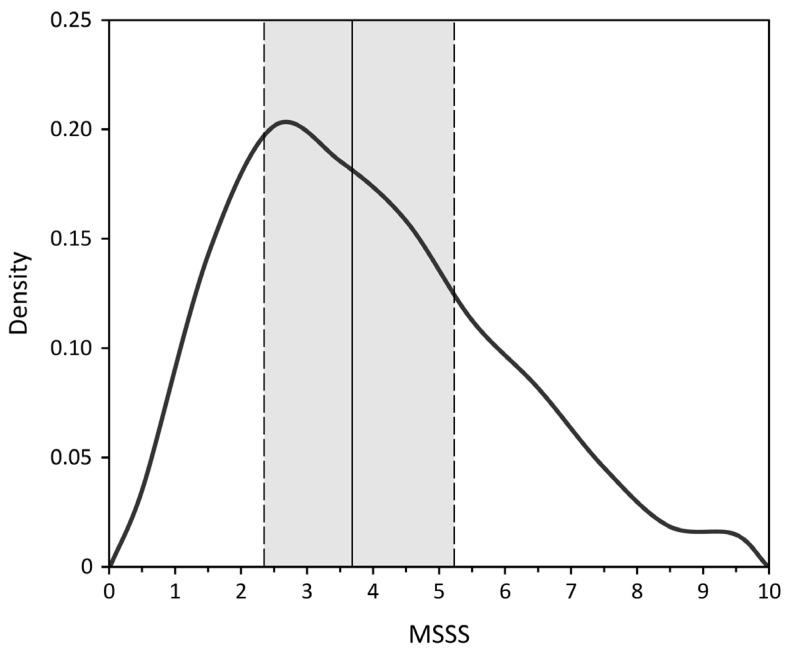
A density plot of the MSSS distribution in the studied sample of MS patients. The solid vertical line indicates median MSSS value; the dashed lines—first and third quartiles (Q_1_ and Q_3_).

MSSS distribution in the studied patients is shown in Figure 1. MSSS values varied from 0.38 to 9.47 with median at 3.69 (the first quartile Q_1_ = 2.34 and the third quartile Q_3_ = 5.24). Based on MSSS distribution we selected thresholds for two binary models: dichotomization (MSSS > 3.5 versus ≤3.5) and extreme phenotype sampling (MSSS > 5.0 versus <2.5). When using median MSSS, 303 patients were compared with 245 patients, and when using extreme MSSS, 150 patients were compared with 150 patients.

### 2.2. Studied Polymorphic Loci, Linkage Disequilibrium, and Statistical Power Calculation

In total, 35 polymorphic loci in 35 genes, *CD58*, *VCAM1*, *EVI5*, *CTLA4*, *CD86*, *EOMES*, *CCR5*, *IL7RA*, *IL4*, *TCF7*, *IL17A*, *HLA-DRB1*, *PSMB9*, *TNF*, *IL22RA2*, *IL6*, *IRF5*, *PVT1*, *IFNB1*, *IL2RA*, *CD6*, *CXCR5*, *TNFRSF1A*, *IFNG*, *CLEC16A*, *SOCS1*, *TNP2*, *IRF8*, *CCL5*, *STAT3*, *TYK2*, *TGFB1*, *CD40*, *IFNAR1*, and *IFNAR2*, were selected for analysis; they are located on 16 chromosomes (Supplementary Table S1). Linkage disequilibrium (LD) analysis was performed for the loci on the same chromosome arm (Supplementary Figure S1). D′ and r^2^ values of LD between most of these loci were low or zero. The exceptions were *TNF* and *HLA-DRB1* (D′ = 42, r^2^ = 7) in the HLA locus on chromosome 6 and the *CLEC16A-SOCS1-TNP2* region on chromosome 16 (D′ range 20 to 75; r^2^ range 2 to 10).

The statistical power of the analysis was estimated using a minor allele frequency (MAF) of 0.3, which corresponds to the mean MAF of the 35 selected polymorphic variants in the European population according to the NCBI SNP database. For the range of effect sizes considered (weak, moderate, and strong, corresponding to OR = 1.5, 2.0, and 2.5, respectively), the mean power across the three genetic models (additive, dominant, recessive) was approximately 80% in the total sample (n = 548), 73% in the female subgroup (n = 387), and 50% in the male subgroup (n = 161). Detailed power calculations for all parameter combinations are provided in Table S2. These estimates indicate that the study was adequately powered to detect the reported associations in the total and female samples, whereas the male subgroup analysis has limited power, particularly for weaker effects.

### 2.3. Association of Immune-Related Gene Variants with MSSS in the Total Sample of MS Patients

When the median MSSS model was considered (Table 1, left panel and Table S3), CXCR5*A, EOMES*T, TNFRSF1A*C, PVT1*G, CCR5*d, IL22RA2*G, and *CLEC16A*-SOCS1*G variants were positively associated with severe MS (*p* = 0.011–0.027; OR = 1.48–1.70), while carriers of the HLA-DRB1*8 allele were enriched in the group of patients with mild MS (*p* = 0.016; OR = 0.50). When the extreme MSSS model was considered, variants CXCR5*A, EOMES*T, IRF8*AA, and PVT1*G were positively associated with severe MS (*p* = 0.0086–0.034; OR = 1.65–2.67) (Table 1, central panel and Table S4). When the continuous MSSS model was considered, CXCR5*A, EOMES*T, TNFRSF1A*C, IRF8*AA and IL6*G variants were positively associated with severe MS (*p* = 0.0037–0.029; absolute β-value = 0.301–0.880) (Table 1, right panel and Table S5). Generally, among the 35 gene polymorphisms studied, five variants, CXCR5*A, EOMES*T, TNFRSF1A*C, IRF8*AA, and PVT1*G, were identified as MSSS-associated in at least two models, and we considered these variants to be modifiers of MS severity. Among them, CXCR5*A and EOMES*T were identified as MSSS-associated loci in all three used models (Table 1).

### 2.4. Association of Immune-Related Gene Variants with MSSS in Female and Male MS Patients

To take into account the possible influence of patients’ sex on the results obtained and to search for possible sex-specific associations, we performed the same association analysis after sex stratification of MS patients (Tables S1–S3). Polymorphic variants associated with MSSS in female and male MS patients are shown in Table 2 and Table 3, respectively.

The analysis of MSSS association with 35 studied polymorphisms in women (Table 2) revealed six variants that were significant in at least two models. Five of them were enriched in patients with severe MS, namely CCR5*d, EOMES*T, HLA-DRB1*15, CXCR5*AA, and IL6*G, while carriers of another *HLA-DRB1* allele, DRB1*8, were enriched in patients with mild MS. CCR5*d and EOMES*T were MSSS-associated in all three models. For male patients, association with MSSS in at least two models was observed for three gene variants (Table 3): CXCR5*A and TCF7*C were identified as markers of more severe MS in all three models, and the CD40*TT variant in two models.

## 3. Discussion

In this retrospective study, we aimed to robustly identify genetic variants associated with MS severity. To achieve this, we applied a comprehensive analytical approach that integrated median-based and extreme-based binary MSSS models alongside treating MSSS as a continuous variable and required consistency across at least two of the three models. This approach allowed us to identify 10 of the 35 analyzed immune-related candidate genes as significantly associated with MSSS in a homogeneous cohort of 548 treatment-naïve Russian MS patients and/or in its subgroups stratified by sex; these genes are shown in Table 4. Among them, the variants in four genes (*CXCR5*, *EOMES*, *CCR5,* and *TCF7*) were significantly associated with MSSS in all three models.

*CXCR5* was associated with MSSS not only in the total sample but also in both female and male MS patients, in a sex-independent manner. This result can be considered a form of internal validation, confirming the observed association in two independent subgroups of the initial sample. The *EOMES* variant identified in the total sample retained its significance in women. Variants in the *CCR5*, *HLA-DRB1*, and *IL6* genes were associated with MSSS only in women, while variants in the *TCF7* and *CD40* genes only in men. Although it is tempting to speculate about the sex specificity of these associations, the obtained data may result, among other factors, from insufficient sample sizes, since the statistical power of the analysis drops significantly after stratification by sex. Our power analysis confirms that while the male subgroup was sufficiently powered to detect large effect sizes, it lacked the power to reliably detect variants with moderate effects. Therefore, the lack of certain associations in men should not be interpreted as definitive evidence of their absence, but rather as a reflection of the limited sample size.

The *CXCR5* gene, for which the most compelling evidence of association with MS severity was obtained (see Table 1, Table 2 and Table 3), encodes the C-X-C motif chemokine receptor 5, the main receptor of T follicular cells that is also present on mature B cells [21]. Recent studies have found that CXCR5 and its main ligand—CXCL13—are implicated in the pathogenesis of several autoimmune diseases including MS [22]. Levels of circulating CXCR5+ T follicular helper (TFH) cells were shown to be elevated in the CNS of MS patients, which suggests the involvement of germinal center (GC) reactions in MS progression [21,23,24]. Interestingly, *PVT1*, another gene associated with MSSS according to our data, encodes a regulatory long non-coding RNA, which promotes the expression of *CXCR5* in a model of spinal cord injury [25]. Our results, together with the investigations cited above, suggest that *CXCR5* may be a promising target for MS therapy.

The *EOMES* gene—for which an association with MS severity was observed across all three models, both in the overall cohort and in the subgroup of women—encodes the transcription factor eomesodermin, highly expressed in effector CD8+ T cells and some CD4+ T cells [26]. CD8+ Tregs have been found to attenuate spontaneous GC reactions, and eomesodermin has been shown to control a molecular program critical for their follicular localization and migration [27]. The studies on the most common MS model—experimental autoimmune encephalomyelitis—have indicated that eomesodermin-expressing T-helper cells are essential for chronic neuroinflammation [28]. Moreover, the number of cytotoxic EOMES+ CD4+ T cells is increased in the peripheral blood and cerebrospinal fluid of patients with secondary progressive MS indicating their involvement in the disease progression [29].

Among other genes associated with MSSS in only one of the three comparisons—whether for the overall group, in female patients, or in male MS patients (see Table 4)—the products of *CCR5* and *IL6* genes affect TFH cell functioning, influencing their migration in the CNS (*CCR5*) [30] or differentiation (*IL6*) [31,32,33]. The *TCF7* gene encodes the transcription factor, which is an important regulator of TFH differentiation [34]. The *CD40* gene encodes the costimulatory protein on the surface of B cells, through which TFH cells provide costimulatory signals essential for a B-cell response [35]. *IRF8* gene product acts as a key molecular switch, regulating the early fate of activated B cells and directing them towards either the GC reaction or rapid differentiation into antibody-secreting plasmablasts [36].

Thus, our findings suggest that MSSS-associated *CXCR5*, *PVT1*, *EOMES*, *CCR5*, *IL6*, *TCF7*, *CD40*, and *IRF8* genes, which, according to the literature, participate in the development of neuroinflammation (namely, in the regulation of TFH activity, formation of GC in the CNS, or B-cell response), may be involved in the progression of MS. In addition, the association of *HLA-DRB1* and *TNFRSF1A* polymorphic variants with MS severity is likely related to broader immune mechanisms, since these genes are key regulators of the immune system essential for MS pathogenesis [37,38].

In general, our data show that MS severity, assessed by the MSSS scale, depends on the combined influence of variants in at least 10 immune-related genes (see Table 4). Some of these genes were studied previously, but did not show significant associations with MS severity [39,40,41,42,43]. This discrepancy in the results obtained across different studies may arise from differences in the size and structure of the studied groups of MS patients, MS course, treatment history, and approaches used to measure MS severity. Indeed, studies [42,43] included small cohorts of ~140–150 MS patients, study [39] combined 10 datasets in one large but heterogeneous sample, work [41] reported that 71% of the cohort was on various treatments at the time of the study, and four other works do not mention treatment status at all. It is tempting to suggest that our positive results are due to an effective study design. The participants were enrolled in the study over a period of about 10 years, which allowed us to carefully select MS patients who were treatment-naïve for various reasons (mild MS, drug unavailability, patient refusal of treatment, etc.) and to measure genetic associations with MS severity without confounding effects of immunomodulatory drugs. In addition, we analyzed an ethnically homogeneous sample of Russian MS patients; all of them had relapsing onset of the disease and were clinically well-characterized at the same medical center, which ensured the homogeneity of the EDSS scores underlying the MSSS scale.

The main limitations of our study should be acknowledged. The ORs for some of the reported associations have wide confidence intervals, primarily due to small sample size and low minor allele frequencies (e.g., MAF ≤ 0.2 for the *IRF8* SNP), which leads to imprecise estimates of the effect sizes. The question of potential sex specificity of several identified genetic associations remains open and needs to be clarified. Thus, our results certainly need to be validated in larger independent cohorts with sufficient statistical power to address these issues and rule out the possibility of false positives, which are common in exploratory genetic association studies. Further functional studies are required to establish solid causal or mechanistic links between the identified polymorphic variants, their host genes, and MS severity.

We believe that broadening research on genetic predictors of MS phenotype may help to clarify the nature of the disease’s clinical heterogeneity and contribute to the development of novel tools for precise disease prognosis.

## 4. Materials and Methods

### 4.1. Patients

A total of 548 unrelated treatment-naive relapsing-onset MS patients diagnosed according to the McDonald criteria (2010 or 2017 revisions) [44,45] were enrolled in the study (387 women, 161 men; female/male ratio 2.4:1). All the MS patients lived in the Moscow region and were followed up at the Moscow City Hospital No. 24. The clinical characteristics of the patients are shown in Table 5; significant differences between women and men were observed only in the median MSSS values (*p* = 0.030).

### 4.2. Genotyping

Genomic DNA was extracted from whole blood using QIAamp Blood Midi Kits (QIAGEN, Hilden, Germany). A brief description of the PCR-based methods used for genotyping of the selected 35 polymorphic variants is provided in Table S1.

### 4.3. Statistics

The Mann–Whitney test was used to evaluate clinical differences between female and male MS patients (*p* < 0.05). LD analysis of polymorphic variants located on the same chromosome arm was carried out using Haploview v.4.2 [46]. The study power was calculated using the genpwr v.3.5.2 [47].

Alleles/genotypes associated with MSSS after dichotomization based on the overall median (median MSSS) or after the extreme phenotype sampling (extreme MSSS) were identified using Fisher’s exact test; carriage of the allele/genotype was considered to be associated with MSSS if Fisher’s exact test *p*-value was <0.05 and the 95% confidence interval (CI) for the odds ratio (OR) did not cross 1. The association with MSSS as a continuous variable (continuous MSSS) was assessed via linear regression using R programming environment v.4.1.3 [48]. If a polymorphic variant was associated with MSSS in at least two of the three used models we considered this variant to be a modifier of MS severity.

## Figures and Tables

**Table 1:** The polymorphic variants associated with MSSS in at least one model—median, extreme or continuous—in the total sample of 548 MS patients.

Carriage of Allele/Genotype	Median MSSS (>3.5 vs. ≤3.5)		Extreme MSSS (>5 vs. <2.5)		Continuous MSSS ^^^	
	*p*-Value	OR [95% CI]	*p*-Value	OR [95% CI]	*p*-Value	β ^#^ (Standard Error)
CXCR5*A	**0.012**	**1.68 [1.09–2.59]**	**0.0086**	**2.20 [1.18–4.09]**	**0.020**	**0.301 (0.129)**
EOMES*T	**0.014**	**1.53 [1.06–2.21]**	**0.023**	**1.73 [1.04–2.88]**	**0.029**	**0.429 (0.195)**
TNFRSF1A*C	**0.024**	**1.50 [1.02–2.19]**	NS	-	**0.0037**	**0.612 (0.210)**
IRF8*AA	NS	-	**0.034**	**2.67 [1.00–7.07]**	**0.015**	**0.880 (0.359)**
PVT1*G	**0.016**	**1.48 [1.05–2.10]**	**0.024**	**1.65 [1.03–2.63]**	NS	-
CCR5*d	0.011	1.70 [1.10–2.62]	NS	**-**	NS	**-**
HLA-DRB1*8 ^$^	0.016	0.50 [0.27–0.91]	NS	**-**	NS	**-**
IL22RA2*G	0.024	1.68 [1.03–2.73]	NS	**-**	NS	**-**
CLEC16A-SOCS1*G	0.027	1.53 [1.01–2.30]	NS	**-**	NS	**-**
IL6*G	NS	-	NS	**-**	0.020	0.302 (0.129)

The asterisk (*) separates the gene name from the allele or genotype designation. The variants associated with MSSS in at least two models are set in bold. The significance of the associations in the median and extreme MSSS models was tested using Fisher’s exact test, and in the continuous MSSS model via linear regression. ^^^ Sex-adjusted; ^$^ variant associated with mild MS; ^#^ absolute value; NS—not significant.

**Table 2:** The polymorphic variants associated with MSSS in at least one model—median, extreme or continuous—in the sample of 387 female MS patients.

Carriage of Allele/Genotype	Median MSSS (>3.5 vs. ≤3.5)		Extreme MSSS (>5 vs. <2.5)		Continuous MSSS	
	*p*-Value	OR [95% CI]	*p*-Value	OR [95% CI]	*p*-Value	β ^#^ (Standard Error)
CCR5*d	**0.0012**	**2.26 [1.35–3.78]**	**0.022**	**2.23 [1.08–4.61]**	**0.045**	**0.488 (0.242)**
EOMES*T	**0.0042**	**1.85 [1.19–2.88]**	**0.0082**	**2.22 [1.19–4.14]**	**0.030**	**0.511 (0.234)**
HLA-DRB1*15	NS	-	**0.027**	**1.79 [1.03–3.11]**	**0.035**	**0.365 (0.173)**
CXCR5*AA	NS	-	**0.021**	**1.89 [1.06–3.45]**	**0.046**	**0.310 (0.155)**
IL6*G	NS	-	**0.024**	**2.04 [1.05–3.97]**	**0.044**	**0.319 (0.158)**
HLA-DRB1*8 ^$^	**0.0067**	**0.39 [0.19–0.80]**	**0.031**	**0.34 [0.12–0.98]**	NS	-
PVT1*G	0.019	1.58 [1.05–2.39]	NS	-	NS	-
IFNG*AT	0.0092	1.66 [1.11–2.48]	NS	-	NS	-
IFNG*A	0.024	1.58 [1.03–2.42]	NS	-	NS	-
EVI5*C	0.028	1.57 [1.01–2.42]	NS	-	NS	-
IFNAR1*G	NS	-	0.022	2.47 [1.08–5.67]	NS	-
TNFRSF1A*C	NS	-	NS	-	0.030	0.542 (0.248)

The asterisk (*) separates the gene name from the allele or genotype designation. The variants associated with MSSS in at least two models are set in bold. The significance of the associations in the median and extreme MSSS models was tested using Fisher’s exact test, and in the continuous MSSS model via linear regression. ^$^ Variant associated with mild MS; ^#^ absolute value; NS—not significant.

**Table 3:** The polymorphic variants associated with MSSS in at least one model—median, extreme or continuous—in the sample of 161 male MS patients.

Carriage of Allele/Genotype	Median MSSS (>3.5 vs. ≤3.5)		Extreme MSSS (>5 vs. <2.5)		Continuous MSSS	
	*p*-Value	OR [95% CI]	*p*-Value	OR [95% CI]	*p*-Value	β ^#^ (Standard Error)
CXCR5*A	**0.00066**	**3.85 [1.74–8.52]**	**0.026**	**3.29 [1.11–9.74]**	**0.026**	**0.597 (0.264)**
TCF7*C	**0.012**	**2.69 [1.18–6.14]**	**0.023**	**4.22 [1.11–16.03]**	**0.034**	**0.756 (0.351)**
CD40*TT	NS	-	**0.0076**	**3.13 [1.32–7.41]**	**0.019**	**0.866 (0.364)**
TNFRSF1A*CT	0.0046	2.48 [1.29–4.78]	NS	-	NS	-
HLA-DRB1*13 ^$^	0.010	0.38 [0.18–0.82]	NS	-	NS	-
TNFRSF1A*C	0.011	2.56 [1.21–5.42]	NS	-	NS	-
*CLEC16A*-SOCS1*G	0.014	2.65 [1.16–6.05]	NS	-	NS	-
CTLA4*A	0.035	2.57 [1.03–6.44]	NS	-	NS	-
IRF8*A	NS	-	NS	-	0.014	0.705 (0.283)
EVI5*C	NS	-	NS	-	0.029	0.802 (0.362)

The asterisk (*) separates the gene name from the allele or genotype designation. The variants associated with MSSS in at least two models are set in bold. The significance of the associations in the median and extreme MSSS models was tested using Fisher’s exact test, and in the continuous MSSS model via linear regression. ^$^ Variant associated with mild MS; ^#^ absolute value; NS—not significant.

**Table 4:** Immune-related genes whose variants are significantly associated with MSSS in at least two of the three models: median, extreme, and continuous.

MSSS-Associated Gene	Total Sample of MS Patients	Female MS Patients	Male MS Patients
*CXCR5*	++	+	++
*EOMES*	++	++	
*TNFRSF1A*	+		
*IRF8*	+		
*PVT1*	+		
*CCR5*		++	
*HLA-DRB1*		+	
*IL6*		+	
*TCF7*			++
*CD40*			+

+ Associations which met our significance criterion: variant is associated with MSSS in at least two models. ++ Associations observed in all three models.

**Table 5:** The clinical data for the treatment-naive multiple sclerosis patients recruited for the study.

Characteristics	Total Sample, n = 548	Women, n = 387	Men, n = 161
MS course (RRMS/SPMS)	504/44	355/32	149/12
		*p* = 0.86	
Median age at onset (range), years	26 (20–34)	27 (21–35)	24 (20–31)
		*p* = 0.053	
Median disease duration before recruitment to the study (range), years	6.0 (3–10)	6 (3–11)	5 (2–10)
		*p* = 0.072	
Median EDSS (range)	2.0 (1.0–6.5)	2.0 (1.0–6.5)	2.0 (1.0–6.0)
		*p* = 0.81	
Median MSSS (range)	3.69 (0.38–9.47)	3.55 (0.38–9.47)	4.27 (0.52–9.08)
		*p* = 0.030	

RRMS—relapsing–remitting multiple sclerosis; SPMS—secondary progressive multiple sclerosis; EDSS—Expanded Disability Status Scale; MSSS—Multiple Sclerosis Severity Score.

## Data Availability

The original contributions presented in this study are included in the article/Supplementary Materials. Further inquiries can be directed to the corresponding author.

## Supplementary Materials

The following supporting information can be downloaded at: https://www.mdpi.com/article/10.3390/ijms27125347/s1. References [49,50,51,52,53,54] are cited in the Supplementary Materials Table S1.

## Abbreviations

The following abbreviations are used in this manuscript:

CI	Confidence interval
CNS	Central nervous system
EDSS	Expanded Disability Status Scale
GC	Germinal center
GWAS	Genome-wide association study
LD	Linkage disequilibrium
MAF	Minor allele frequency
MS	Multiple sclerosis
MSSS	Multiple Sclerosis Severity Score
OR	Odds ratio
TFH	T follicular helper cells
