# Supplementary material for: Immune-Related Gene Variants as Modifiers of Multiple Sclerosis Severity
Source: Int J Mol Sci. 2026 Jun 13;27(12):5347. doi: 10.3390/ijms27125347 (PMC13299223; doi:10.3390/ijms27125347)
Supplement: Supplementary file 1 [file ijms-27-05347-s001.zip › ijms-4335705-supplementary.pdf]

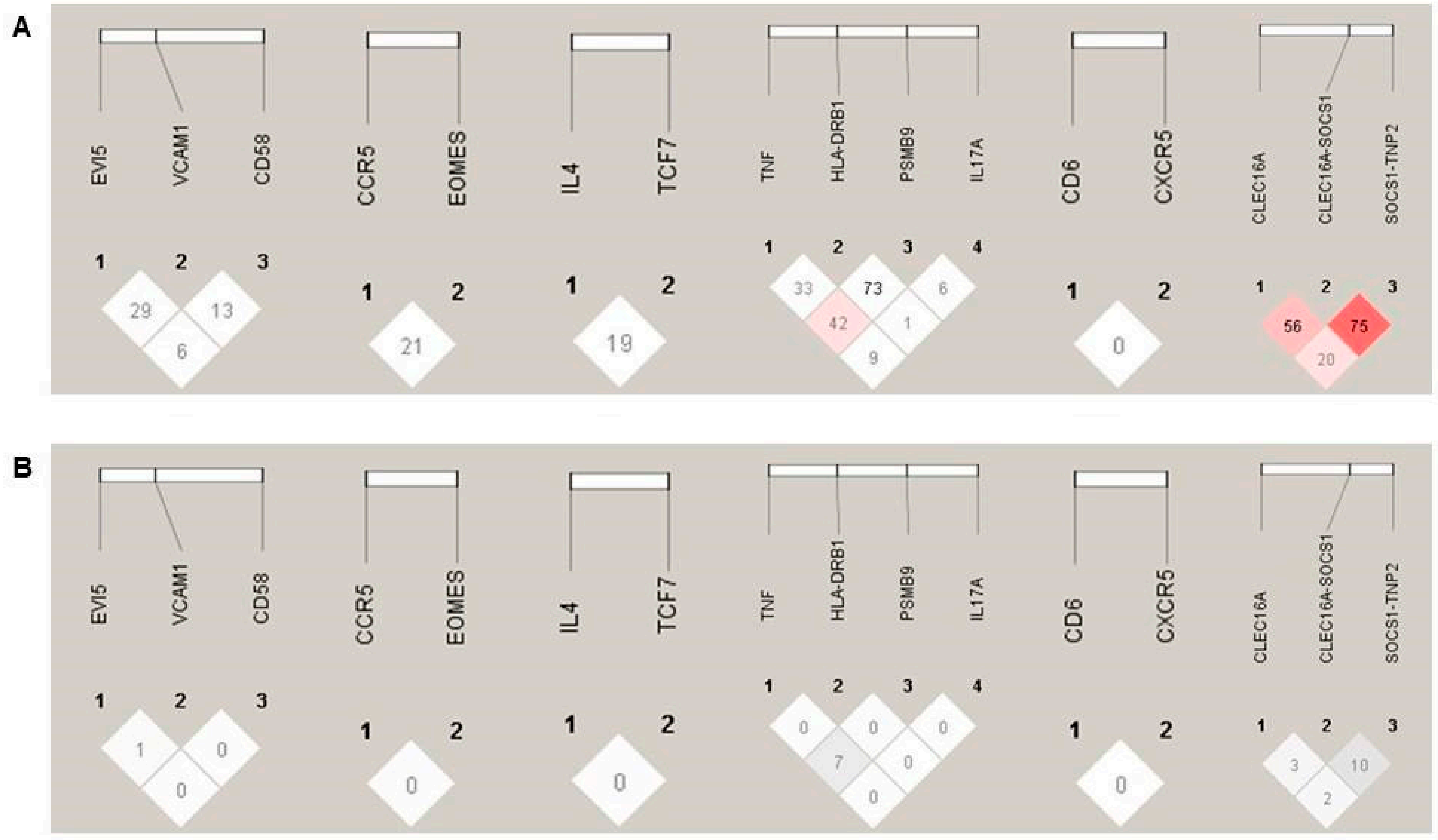

Figure S1. Plots representing D` (A) and r^2 (B) values obtained in the linkage disequilibrium analysis for all loci on the same chromosome arms.

Table S1. Genes and their polymorphisms selected for analysis.

Chromo-some	Locus	Gene	Gene product	SNP ID	Allele 1/ Allele 2	Genotyping method or reference
1	1p13.1	*CD58*	Lymphocyte function-associated antigen 3 (LFA-3)	rs2300747	A/G	[Bashinskaya et al. 2015]
	1p21.2	*VCAM1*	Vascular cell adhesion molecule 1	rs7552544	C/T	TaqMan genotyping assay (Applied Biosystems, Foster City, CA, USA)
	1p22.1	*EVI5*	Ecotropic viral integration site 5 protein homolog	rs11804321	C/T	TaqMan genotyping assay (Applied Biosystems, Foster City, CA, USA)
2	2q33.2	*CTLA4*	Cytotoxic T-lymphocyte antigen 4	rs231775	A/G	[Makarycheva et al. 2011]
3	3p21.31	*CD86*	Cluster of Differentiation 86	rs2255214	G/T	TaqMan genotyping assay (Applied Biosystems, Foster City, CA, USA)
	3p24.1	*EOMES* (downstream)	Eomesodermin (T-box brain protein 2)	rs2371108	G/T	TaqMan genotyping assay (Applied Biosystems, Foster City, CA, USA)
	3q13.33	*CCR5*	C-C chemokine receptor type 5	rs333	w/d	[Makarycheva et al. 2011]
5	5p13.2	*IL7RA*	Interleukin-7 receptor subunit alpha	rs6897932	C/T	[Bashinskaya et al. 2015]
	5q31.1	*IL4*	Interleukin 4	rs2243250	C/T	[Makarycheva et al. 2011]
	5q31.1	*TCF7*	Transcription Factor 7 (T-Cell Specific, HMG-Box)	rs756699	C/T	TaqMan genotyping assay (Applied Biosystems, Foster City, CA, USA)
6	6p12.2	*IL17A*	Interleukin-17A	rs227519	A/G	TaqMan genotyping assay (Applied Biosystems, Foster City, CA, USA)
	6p21.32	*HLA-DRB1*	HLA class II histocompatibility antigen, DRB1 beta chain	Groups of alleles corresponding to the specificities from DR1 to DR18 (low-resolution genotyping)		[Tsareva et al. 2012]
	6p21.32	*PSMB9*	Proteasome subunit beta type-9	rs17587	A/G	TaqMan genotyping assay (Applied Biosystems, Foster City, CA, USA)
	6p21.33	*TNF*	Tumor necrosis factor [alpha]	rs1800629	A/G	[He et al. 1995 8550811]
	6q23.3	*IL22RA2*	Interleukin 22-binding protein	rs202573	A/G	TaqMan genotyping assay (Applied Biosystems, Foster City, CA, USA)
7	7p15.3	*IL6*	Interleukin 6	rs1800795	C/G	[Kiselev et al. 2019]
	7q32.1	*IRF5*	Interferon regulatory factor 5	rs4728142	A/G	TaqMan genotyping assay (Applied Biosystems, Foster City, CA, USA)
8	8q24.21	*PVT1*	PVT1 long non-coding RNA	rs2114358	A/G	TaqMan genotyping assay (Applied Biosystems, Foster City, CA, USA)
9	9p21.3	*IFNB1*	Interferon beta 1	rs1051922	C/T	[Tsareva et al. 2012]
10	10p15.1	*IL2RA*	Interleukin 2 receptor subunit alpha	rs2104286	A/G	[Bashinskaya et al. 2015]
11	11q12.2	*CD6*	T-cell differentiation antigen CD6	rs17824933	C/G	[Bashinskaya et al. 2015]
	11q23.3	*CXCR5*	C-X-C chemokine receptor type 5	rs523604	A/G	TaqMan genotyping assay (Applied Biosystems, Foster City, CA, USA)

12	12p13.31	*TNFRSF1A*	TNF Receptor I	rs1800693	C/T	[Bashinskaya et al. 2015]
	12q15	*IFNG*	Interferon gamma	rs2430561	A/T	[Pravica et al. 2000 11053629]
16	16p13.13	*CLEC16A*	CLEC16A: C-Type Lectin Domain Family 16 Member A	rs6498169	A/G	[Bashinskaya et al. 2015]
	16p13.13	*CLEC16A-SOCS1 (intergenic)*	SOCS1: Suppressor of cytokine signaling 1	rs1640923	A/G	PCR-RFLP<br>A 357-bp fragment was amplified with primers: 5`- CACTCTGATGAACTCCCCAACA-3` and 5`- GTCAGTGACCAGGAACTGGAAG-3` and cut if containing G allele with restriction endonuclease *Bse1I* ("Sybenzyme", Russia)
	16p13.13	*SOCS1-TNP2 (intergenic)*	TNP2: Transition Protein 2	rs243324	C/T	TaqMan genotyping assay (Applied Biosystems, Foster City, CA, USA)
	16q24.1	*IRF8*	Interferon regulatory factor 8	rs17445836	A/G	TaqMan genotyping assay (Applied Biosystems, Foster City, CA, USA)
17	17q12	*CCL5*	C-C Chemokine Ligand 5	rs2107538	A/G	[Kiselev et al. 2019]
	17q21.2	*STAT3*	Signal transducer and activator of transcription 3	rs744166	C/T	[Bashinskaya et al. 2015]
19	19p13.2	*TYK2*	Non-receptor tyrosine-protein kinase TYK2	rs2304256	A/C	TaqMan genotyping assay (Applied Biosystems, Foster City, CA, USA)
	19q13.2	*TGFB1*	Transforming growth factor beta 1	rs1800469	C/T	[Makarycheva et al. 2011]
20		*CD40*	Cluster of differentiation 40 (TNFSF5)	rs6074022	C/T	TaqMan genotyping assay (Applied Biosystems, Foster City, CA, USA)
21	21q22.11	*IFNAR1*	Interferon-alpha/beta receptor alpha chain	rs1012335	C/G	[Tsareva et al. 2012]
	21q22.11	*IFNAR2*	Interferon-alpha/beta receptor beta chain	rs2248202	A/C	TaqMan genotyping assay (Applied Biosystems, Foster City, CA, USA)

References

1. Bashinskaya VV, Kulakova OG, Kiselev IS, Baulina NM, Favorov AV, Boyko AN, Tsareva EY, Favorova OO. GWAS-identified multiple sclerosis risk loci involved in immune response: validation in Russians. J Neuroimmunol. 2015 May 15;282:85-91. doi: 10.1016/j.jneuroim.2015.03.015.
2. Makarycheva OY, Tsareva EY, Sudomoina MA, Kulakova OG, Titov BV, Bykova OV, Gol'tsova NV, Kuzenkova LM, Boiko AN, Favorova OO. Family Analysis of Linkage and Association of HLA-DRB1, CTLA4, TGFB1, IL4, CCR5, RANTES, MMP9 and TIMP1 Gene Polymorphisms with Multiple Sclerosis. Acta Naturae. 2011 Jan;3(1):85-92.
3. Tsareva EY, Kulakova OG, Boyko AN, Shchur SG, Lvovs D, Favorov AV, Gusev EI, Vandenbroeck K, Favorova OO. Allelic combinations of immune-response genes associated with glatiramer acetate treatment response in Russian multiple sclerosis patients. Pharmacogenomics. 2012 Jan;13(1):43-53. doi: 10.2217/pgs.11.136.
4. He B, Navikas V, Lundahl J, Söderström M, Hillert J. Tumor necrosis factor alpha-308 alleles in multiple sclerosis and optic neuritis J Neuroimmunol . 1995 Dec 31;63(2):143-7. doi: 10.1016/0165-5728(95)00138-7.
5. Kiselev I, Bashinskaya V, Baulina N, Kozin M, Popova E, Boyko A, Favorova O, Kulakova O. Genetic differences between primary progressive and relapsing-remitting multiple sclerosis: The impact of immune-related genes variability. Mult Scler Relat Disord. 2019 Apr;29:130-136. doi: 10.1016/j.msard.2019.01.033.
6. Pravica V, Perrey C, Stevens A, Lee J H, Hutchinson I V. A single nucleotide polymorphism in the first intron of the human IFN-gamma gene: absolute correlation with a polymorphic CA microsatellite marker of high IFN-gamma production Hum Immunol . 2000 Sep;61(9):863-6. doi: 10.1016/s0198-8859(00)00167-1. PMID: 11053629 DOI: 10.1016/s0198-8859(00)00167-1.

Table S2. Results of the statistical power analysis for a minor allele frequency (MAF) of 0.3

Sample	OR	Model	Power at Alpha = 0.05	Average power
All patients (n = 548)	2.5	Dominant	1.00	0.80
		Recessive	0.84	
		Additive	1.00	
	2	Dominant	0.98	
		Recessive	0.62	
		Additive	1.00	
	1.5	Dominant	0.66	
		Recessive	0.27	
		Additive	0.86	
Women (n = 387)	2.5	Dominant	0.99	0.73
		Recessive	0.70	
		Additive	1.00	
	2	Dominant	0.92	
		Recessive	0.48	
		Additive	0.99	
	1.5	Dominant	0.51	
		Recessive	0.21	
		Additive	0.73	
Men (n = 161)	2.5	Dominant	0.82	0.50
		Recessive	0.36	
		Additive	0.95	
	2	Dominant	0.59	
		Recessive	0.23	
		Additive	0.79	
	1.5	Dominant	0.25	
		Recessive	0.11	
		Additive	0.38	

Table S3. Alleles/genotypes of immune-related genes. associated with relatively severe MS course (MSSS >3.5) (median MSSS)

Carriage of alleles/genotypes	Carriers (%). patients with MSSS >3.5	Carriers (%). patients with MSSS ≤3.5	p_f values	OR [95%CI]
All MS patients (n=548)				
*CCR5**d	72 (23.8)	38 (15.6)	0.011	1.70 [1.10-2.62]
*CXCR5**A	255 (84.7)	188 (76.7)	0.012	1.68 [1.09-2.59]
*EOMES**T	218 (72.9)	151 (63.7)	0.014	1.53 [1.06-2.21]
*HLA-DRB1**8^	19 (6.3)	29 (11.9)	0.016	0.50 [0.27-0.91]
*PVT1**G	196 (65.1)	136 (55.7)	0.016	1.48 [1.05-2.10]
*IL22RA2**G	268 (88.7)	202 (82.4)	0.024	1.68 [1.03-2.73]
*TNFRSF1A**CT	162 (53.5)	105 (42.9)	0.0085	1.53 [1.09-2.15]
*TNFRSF1A**C	234 (77.2)	170 (69.4)	0.024	1.50 [1.02-2.19]
*CLEC16A-SOCS1**G	79 (26.1)	46 (18.8)	0.027	1.53 [1.01-2.30]
Women (n=387)				
*CCR5**d	56 (27.3)	26 (14.3)	0.0012	2.26 [1.35-3.78]
*EOMES**GT	117 (57.6)	73 (41.7)	0.0014	1.90 [1.26-2.86]
*EOMES**T	153 (75.4)	109 (62.3)	0.0042	1.85 [1.19-2.88]
*HLA-DRB1**8^	12 (5.9)	25 (13.8)	0.0067	0.39 [0.19-0.80]
*PVT1**G	136 (66.3)	101 (55.5)	0.019	1.58 [1.05-2.39]
*IFNG**A/T	113 (55.7)	78 (43.1)	0.0092	1.66 [1.11-2.48]
*IFNG**A	146 (71.9)	112 (61.9)	0.024	1.58 [1.03-2.42]
*EVI5**C	74 (36.1)	48 (26.5)	0.028	1.57 [1.01-2.42]
Men (n=161)				
*CXCR5**A	86 (87.8)	41 (65.1)	0.00066	3.85 [1.74-8.52]
*TNFRSF1A**C/T	56 (57.1)	22 (34.9)	0.0046	2.48 [1.29-4.78]
*HLA-DRB1**13^	15 (15.3)	20 (32.3)	0.010	0.38 [0.18-0.82]
*TNFRSF1A**C	82 (83.7)	42 (66.7)	0.011	2.56 [1.21-5.42]
*TCF7**C	30 (30.9)	9 (14.3)	0.012	2.69 [1.18-6.14]
*CLEC16A-SOCS1**G	30 (30.6)	9 (14.3)	0.014	2.65 [1.16-6.05]
*CTLA4**A	89 (90.8)	50 (79.4)	0.035	2.57 [1.03-6.44]

^Allele associated with mild MS course (MSSS≤3.5).

Table S4. Alleles/genotypes of immune-related genes. associated with severe MS course (MSSS > 5) (extreme MSSS)

Carriage of alleles/genotypes	Carriers (%). patients with MSSS >5	Carriers (%). patients with MSSS <2.5	p_f values	OR [95%CI]
All MS patients (n=300)				
*CXCR5**A	130 (86.7)	115 (76.7)	0.0086	2.20 [1.18-4.09]
*CXCR5**AA	59 (39.3)	41 (27.3)	0.015	1.76 [1.08-2.87]
*EOMES**T	115 (76.7)	95 (63.3)	0.023	1.73 [1.04-2.88]
*PVT1**G	98 (65.3)	81 (54.0)	0.024	1.65 [1.03-2.63]
*IRF8**AA	15 (10.0)	6 (4.0)	0.034	2.67 [1.00-7.07]
Women (n=207)				
*EOMES**GT	61 (62.2)	44 (41.5)	0.0023	2.32 [1.32-4.08]
*EOMES**T	77 (78.6)	66 (62.3)	0.0082	2.22 [1.19-4.14]
*CXCR5**AA	40 (41.7)	30 (27.3)	0.021	1.89 [1.06-3.45]
*CCR5**d	24 (24.7)	14 (12.8)	0.022	2.23 [1.08-4.61]
*IFNAR1**G	88 (90.7)	87 (79.8)	0.022	2.47 [1.08-5.67]
*IL6**G	80 (82.5)	76 (69.7)	0.024	2.04 [1.05-3.97]
*DRB1**15	54 (55.7)	45 (41.3)	0.027	1.79 [1.03-3.11]
*DRB1**8^	5 (5.2)	15 (13.8)	0.031	0.34 [0.12-0.98]
Men (n=93)				
*CD40**TT	32 (62.7)	14 (35.0)	0.0076	3.13 [1.32-7.41]
*TCF7**C	13 (25.5)	3 (7.5)	0.023	4.22 [1.11-16.03]
*CXCR5**A	46 (88.5)	28 (70.0)	0.026	3.29 [1.11-9.74]

^Allele associated with mild MS course (MSSS<2.5).

Table S5. Genotypes of immune-related genes. associated with MSSS value (continuous MSSS)

Model	Gene	Polymorphism ID	Allele 1/ Allele 2	β (Allele2)	Std. Error	p-value
All MS patients (n=548)						
Additive model (A1A1 vs A1A2 vs A2A2)	*CXCR5*	rs523604	A/G	-0.301	0.129	0.020
	TNFRSF1A	rs1800693	C/T	-0.307	0.128	0.017
	IL6	rs1800795	C/G	0.302	0.129	0.020
	IRF8	rs17445836	A/G	-0.314	0.145	0.031
Recessive model (A1A1 vs A1A2 + A2A2)	*IRF8*	rs17445836	A/G	-0.880	0.359	0.015
	EOMES	rs2371108	G/T	0.429	0.195	0.029
Dominant model (A1A1 + A1A2 vs A2A2)	*TNFRSF1A*	rs1800693	C/T	-0.612	0.210	0.0037
	CXCR5	rs523604	A/G	-0.548	0.237	0.021
Women (n=387)						
Additive model (A1A1 vs A1A2 vs A2A2)	CCR5	rs333	w/d	0.488	0.242	0.045
	CXCR5	rs523604	A/G	-0.310	0.155	0.046
	IL6	rs1800795	C/G	0.319	0.158	0.044
	TNFRSF1A	rs1800693	C/T	0.299	0.152	0.049
	*DRB1**15			0.365	0.173	0.035
Recessive model (A1A1 vs A1A2 + A2A2)	*EOMES*	rs2371108	G/T	0.511	0.234	0.030
Dominant model (A1A1 + A1A2 vs A2A2)	*TNFRSF1A*	rs1800693	C/T	0.542	0.248	0.030
	DRB1	-	15/other	0.400	0.202	0.049
Men (n=161)						
Additive model (A1A1 vs A1A2 vs A2A2)	*CXCR5*	rs523604	A/G	-0.597	0.264	0.026
	IRF8	rs17445836	A/G	-0.705	0.283	0.014
	EVI5	rs11804321	T/C	0.802	0.362	0.029
	TCF7	rs756699	T/C	-0.756	0.351	0.034
Dominant model (A1A1 + A1A2 vs A2A2)	*IRF8*	rs17445836	A/G	-0.756	0.375	0.047
	CD40	rs6074022	T/C	0.866	0.364	0.019
	EVI5	rs11804321	T/C	0.894	0.422	0.036
